# (2*E*)-3-(3-Bromo-4-meth­oxy­phen­yl)-1-(4,4′′-difluoro-5′-meth­oxy-1,1′:3′,1′′-terphenyl-4′-yl)prop-2-en-1-one

**DOI:** 10.1107/S1600536811050884

**Published:** 2011-11-30

**Authors:** Hoong-Kun Fun, Tara Shahani, S. Samshuddin, B. Narayana, B. K. Sarojini

**Affiliations:** a X-ray Crystallography Unit, School of Physics, Universiti Sains Malaysia, 11800 USM, Penang, Malaysia; b Department of Studies in Chemistry, Mangalore University, Mangalagangotri 574 199, India; c Department of Chemistry, P.A. College of Engineering, Nadupadavu, Mangalore 574 153, India

## Abstract

In the title compound, C_29_H_21_BrF_2_O_3_, the dihedral angles between the central anisole ring and the pendant fluoro­benzene rings are 48.86 (19) and 31.89 (18)°. The dihedral angle between the anisole ring and the 1-bromo-2-meth­oxy­benzene ring linked *via* the enone bridge is 82.95 (17)°. In the crystal, C—H⋯O hydrogen bonds link the mol­ecules into *C*(11) chains propagating along [010].

## Related literature

For related structures and background to chalcones and their properties, see: Fun *et al.* (2010**a* ▶,b* ▶).
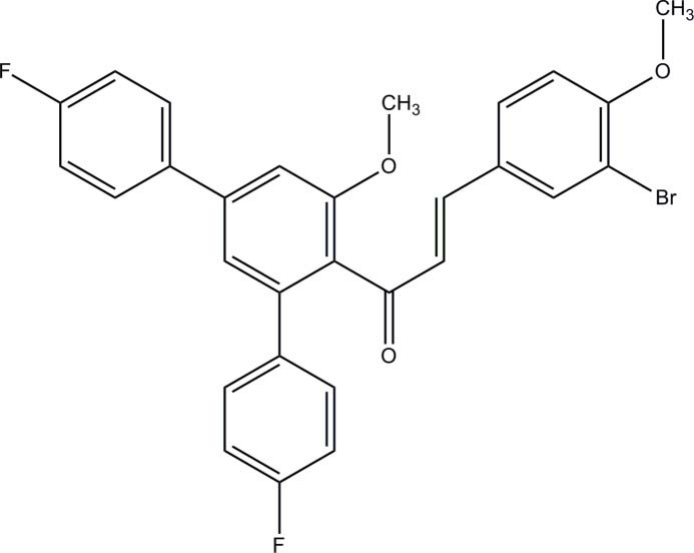


## Experimental

### 

#### Crystal data

C_29_H_21_BrF_2_O_3_
*M* *_r_* = 535.37
Monoclinic, 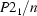

*a* = 9.6902 (6) Å
*b* = 20.3345 (12) Å
*c* = 12.9556 (8) Å
β = 110.636 (1)°
*V* = 2389.0 (3) Å^3^
*Z* = 4
Mo *K*α radiation
μ = 1.77 mm^−1^
*T* = 296 K
0.42 × 0.15 × 0.10 mm

#### Data collection

Bruker SMART APEXII CCD diffractometer
Absorption correction: multi-scan (*SADABS*; Bruker, 2009) ▶ *T*_min_ = 0.525, *T*_max_ = 0.843
22747 measured reflections
5455 independent reflections
4111 reflections with *I* > 2σ(*I*)
*R*_int_ = 0.032

#### Refinement

*R*[*F* ^2^ > 2σ(*F* ^2^)] = 0.056
*wR*(*F* ^2^) = 0.183
*S* = 1.04
5455 reflections
318 parameters
H-atom parameters constrained
Δρ_max_ = 1.96 e Å^−3^
Δρ_min_ = −0.39 e Å^−3^

### 

Data collection: *APEX2* (Bruker, 2009 ▶); cell refinement: *SAINT* (Bruker, 2009 ▶); data reduction: *SAINT*; program(s) used to solve structure: *SHELXTL* (Sheldrick, 2008 ▶); program(s) used to refine structure: *SHELXTL*; molecular graphics: *SHELXTL*; software used to prepare material for publication: *SHELXTL* and *PLATON* (Spek, 2009 ▶).

## Supplementary Material

Crystal structure: contains datablock(s) global, I. DOI: 10.1107/S1600536811050884/hb6536sup1.cif

Structure factors: contains datablock(s) I. DOI: 10.1107/S1600536811050884/hb6536Isup2.hkl

Supplementary material file. DOI: 10.1107/S1600536811050884/hb6536Isup3.cml

Additional supplementary materials:  crystallographic information; 3D view; checkCIF report

## Figures and Tables

**Table 1:** Hydrogen-bond geometry (Å, °)

*D*—H⋯*A*	*D*—H	H⋯*A*	*D*⋯*A*	*D*—H⋯*A*
C29—H29*B*⋯O2^i^	0.96	2.41	3.303 (6)	155

Symmetry code: (i) 
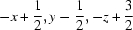
.

**Fig. 1.**
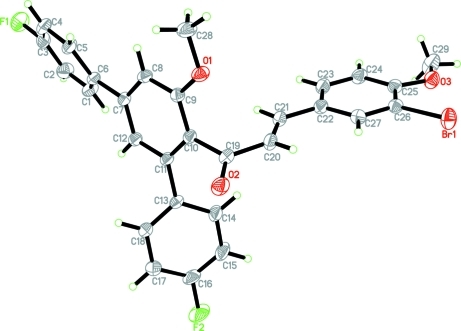
The molecular structure of the title compound showing 30% probability displacement ellipsoids.

**Fig. 2.**
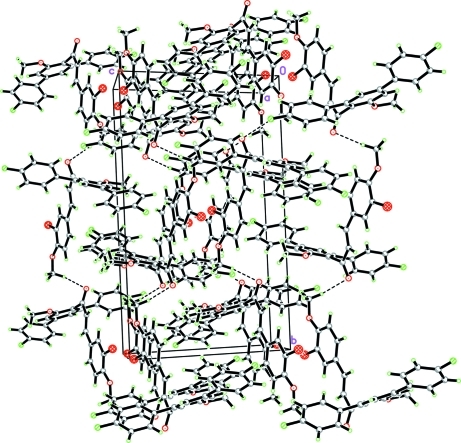
The crystal packing of the title compound, showing chains along [010].

## supplementary crystallographic information

### Comment

As part of our ongoing studies of substituted chalcone derivatives, (Fun *et al.*, 2010**a*,b*), the title compound (I) was prepared and its crystal structure is reported. The precursor of the title compound was prepared from 4,4'-difluoro chalcone by several steps.

The title molecule is built up (Fig. 1) from four units, namely: two fluorobenzenes (C1–C6/F1) and (C13–C18/F2), a anisole (C7–C12/O1/C28) and a 1-bromo-2- methoxybenzene (C22/C27/Br1/C29/O3). The anisole moiety makes dihedral angles of 48.86 (19)°, 31.89 (18)° and 82.95 (17)° with the two fluorobenzenes and 1-bromo-2-methoxybenzene moieties respectively.

In the crystal (Fig. 2), C29—H29B···O2 hydrogen bonds link the molecules into chains along [010].

### Experimental

To a mixture of 1-(4,4''-difluoro-5'-methoxy-1,1':3',1''-terphenyl-4'-yl) ethanone (0.338 g, 0.001 mol) and 3-bromo-4-methoxybenzaldehyde (0.215 g, 0.001 mol) in 30 ml e thanol, 0.5 ml of 10% sodium hydroxide solution was added and stirred at 5–10 °C for 3 h. The precipitate formed was collected by filtration and purified by recrystallization from ethanol. Colourless blocks of (I) were grown from DMF by slow evaporation and the yield of the compound was 82%. *Mp*: 452 K.

### Refinement

H atoms were positioned geometrically [C–H = 0.9300 or 0.9600 Å] and refined using a riding model, with *U*_iso_(H) = 1.2 *U*_eq_ (C) or 1.5*U*_iso_(C).

### Crystal data

**Table:**

C_29_H_21_BrF_2_O_3_	*F*(000) = 1088
*M**_r_* = 535.37	*D*_x_ = 1.488 Mg m^−^^3^
Monoclinic, *P*2_1_/*n*	Mo *K*α radiation, λ = 0.71073 Å
Hall symbol: -P 2yn	Cell parameters from 5893 reflections
*a* = 9.6902 (6) Å	θ = 2.3–26.7°
*b* = 20.3345 (12) Å	µ = 1.77 mm^−^^1^
*c* = 12.9556 (8) Å	*T* = 296 K
β = 110.636 (1)°	Block, colourless
*V* = 2389.0 (3) Å^3^	0.42 × 0.15 × 0.10 mm
*Z* = 4	

### Data collection

**Table:**

Bruker SMART APEXII CCD diffractometer	5455 independent reflections
Radiation source: fine-focus sealed tube	4111 reflections with *I* > 2σ(*I*)
graphite	*R*_int_ = 0.032
φ and ω scans	θ_max_ = 27.5°, θ_min_ = 2.0°
Absorption correction: multi-scan (*SADABS*; Bruker, 2009)	*h* = −12→12
*T*_min_ = 0.525, *T*_max_ = 0.843	*k* = −26→26
22747 measured reflections	*l* = −16→16

### Refinement

**Table:**

Refinement on *F*^2^	Primary atom site location: structure-invariant direct methods
Least-squares matrix: full	Secondary atom site location: difference Fourier map
*R*[*F*^2^ > 2σ(*F*^2^)] = 0.056	Hydrogen site location: inferred from neighbouring sites
*wR*(*F*^2^) = 0.183	H-atom parameters constrained
*S* = 1.04	*w* = 1/[σ^2^(*F*_o_^2^) + (0.1002*P*)^2^ + 2.471*P*] where *P* = (*F*_o_^2^ + 2*F*_c_^2^)/3
5455 reflections	(Δ/σ)_max_ < 0.001
318 parameters	Δρ_max_ = 1.96 e Å^−^^3^
0 restraints	Δρ_min_ = −0.39 e Å^−^^3^

### Special details

**Table:**

Geometry. All e.s.d.'s (except the e.s.d. in the dihedral angle between two l.s. planes) are estimated using the full covariance matrix. The cell e.s.d.'s are taken into account individually in the estimation of e.s.d.'s in distances, angles and torsion angles; correlations between e.s.d.'s in cell parameters are only used when they are defined by crystal symmetry. An approximate (isotropic) treatment of cell e.s.d.'s is used for estimating e.s.d.'s involving l.s. planes.
Refinement. Refinement of *F*^2^ against ALL reflections. The weighted *R*-factor *wR* and goodness of fit *S* are based on *F*^2^, conventional *R*-factors *R* are based on *F*, with *F* set to zero for negative *F*^2^. The threshold expression of *F*^2^ > σ(*F*^2^) is used only for calculating *R*-factors(gt) *etc*. and is not relevant to the choice of reflections for refinement. *R*-factors based on *F*^2^ are statistically about twice as large as those based on *F*, and *R*- factors based on ALL data will be even larger.

### Fractional atomic coordinates and isotropic or equivalent isotropic displacement parameters (Å^2^)

**Table:**

	*x*	*y*	*z*	*U*_iso_*/*U*_eq_	
Br1	0.78171 (4)	0.01704 (2)	0.94057 (4)	0.05838 (18)	
F1	−0.8898 (3)	0.02285 (16)	0.3378 (3)	0.0829 (9)	
F2	−0.0561 (4)	0.2180 (2)	1.2026 (2)	0.0959 (11)	
O1	0.0167 (3)	0.16491 (14)	0.5498 (2)	0.0466 (6)	
O2	0.1325 (3)	0.24970 (13)	0.7986 (2)	0.0544 (7)	
O3	0.6494 (3)	−0.11520 (13)	0.9196 (2)	0.0549 (7)	
C1	−0.5661 (4)	0.0674 (2)	0.5680 (3)	0.0491 (9)	
H1A	−0.5261	0.0601	0.6436	0.059*	
C2	−0.7014 (5)	0.0404 (2)	0.5087 (4)	0.0576 (10)	
H2A	−0.7535	0.0160	0.5435	0.069*	
C3	−0.7566 (4)	0.0505 (2)	0.3978 (4)	0.0556 (10)	
C4	−0.6843 (5)	0.0873 (2)	0.3442 (3)	0.0593 (11)	
H4A	−0.7248	0.0932	0.2683	0.071*	
C5	−0.5502 (4)	0.1154 (2)	0.4048 (3)	0.0501 (9)	
H5A	−0.5012	0.1413	0.3695	0.060*	
C6	−0.4877 (4)	0.10529 (17)	0.5181 (3)	0.0377 (7)	
C7	−0.3388 (4)	0.13085 (16)	0.5830 (3)	0.0359 (7)	
C8	−0.2327 (4)	0.13664 (16)	0.5331 (3)	0.0374 (7)	
H8A	−0.2575	0.1274	0.4585	0.045*	
C9	−0.0913 (4)	0.15609 (16)	0.5949 (3)	0.0352 (7)	
C10	−0.0501 (4)	0.17015 (14)	0.7068 (3)	0.0334 (6)	
C11	−0.1565 (4)	0.16733 (15)	0.7562 (3)	0.0343 (7)	
C12	−0.2992 (4)	0.14751 (16)	0.6934 (3)	0.0370 (7)	
H12A	−0.3700	0.1454	0.7265	0.044*	
C13	−0.1240 (4)	0.18309 (16)	0.8754 (3)	0.0358 (7)	
C14	−0.0050 (4)	0.1557 (2)	0.9580 (3)	0.0498 (9)	
H14A	0.0608	0.1290	0.9396	0.060*	
C15	0.0168 (5)	0.1677 (3)	1.0683 (3)	0.0655 (13)	
H15A	0.0965	0.1490	1.1238	0.079*	
C16	−0.0789 (5)	0.2065 (3)	1.0936 (3)	0.0594 (11)	
C17	−0.1966 (5)	0.2347 (2)	1.0165 (3)	0.0594 (11)	
H17A	−0.2609	0.2615	1.0366	0.071*	
C18	−0.2189 (4)	0.2226 (2)	0.9060 (3)	0.0501 (9)	
H18A	−0.2995	0.2414	0.8517	0.060*	
C19	0.1073 (4)	0.19288 (17)	0.7681 (3)	0.0387 (7)	
C20	0.2285 (4)	0.14534 (19)	0.7912 (3)	0.0448 (8)	
H20A	0.3242	0.1617	0.8134	0.054*	
C21	0.2106 (4)	0.08060 (18)	0.7826 (3)	0.0417 (8)	
H21A	0.1137	0.0658	0.7531	0.050*	
C22	0.3256 (4)	0.02905 (18)	0.8141 (3)	0.0409 (8)	
C23	0.2855 (5)	−0.03641 (19)	0.8042 (3)	0.0484 (9)	
H23A	0.1861	−0.0475	0.7752	0.058*	
C24	0.3908 (4)	−0.08650 (18)	0.8367 (3)	0.0489 (9)	
H24A	0.3613	−0.1303	0.8284	0.059*	
C25	0.5372 (4)	−0.07106 (17)	0.8808 (3)	0.0421 (8)	
C26	0.5802 (4)	−0.00506 (17)	0.8881 (3)	0.0383 (7)	
C27	0.4753 (4)	0.04443 (16)	0.8566 (3)	0.0400 (7)	
H27A	0.5050	0.0882	0.8638	0.048*	
C28	−0.0242 (5)	0.1550 (3)	0.4338 (3)	0.0589 (11)	
H28A	0.0605	0.1613	0.4128	0.088*	
H28B	−0.0993	0.1861	0.3953	0.088*	
H28C	−0.0611	0.1112	0.4154	0.088*	
C29	0.6106 (6)	−0.1824 (2)	0.9226 (5)	0.0765 (16)	
H29A	0.6980	−0.2078	0.9582	0.115*	
H29B	0.5628	−0.1982	0.8487	0.115*	
H29D	0.5447	−0.1866	0.9629	0.115*	

### Atomic displacement parameters (Å^2^)

**Table:**

	*U*^11^	*U*^22^	*U*^33^	*U*^12^	*U*^13^	*U*^23^
Br1	0.0392 (3)	0.0565 (3)	0.0762 (3)	−0.00361 (17)	0.0164 (2)	0.00561 (19)
F1	0.0413 (15)	0.103 (2)	0.084 (2)	−0.0160 (14)	−0.0029 (13)	−0.0303 (17)
F2	0.085 (2)	0.171 (3)	0.0257 (12)	0.012 (2)	0.0129 (12)	−0.0121 (16)
O1	0.0403 (14)	0.0676 (16)	0.0333 (12)	−0.0086 (12)	0.0147 (10)	−0.0034 (11)
O2	0.0516 (16)	0.0418 (13)	0.0579 (17)	−0.0058 (12)	0.0043 (13)	−0.0058 (12)
O3	0.0495 (16)	0.0413 (13)	0.0627 (18)	0.0056 (12)	0.0056 (13)	0.0002 (12)
C1	0.045 (2)	0.062 (2)	0.0364 (18)	−0.0089 (17)	0.0105 (16)	−0.0036 (17)
C2	0.047 (2)	0.069 (2)	0.056 (2)	−0.014 (2)	0.0173 (19)	−0.010 (2)
C3	0.0277 (18)	0.067 (3)	0.061 (3)	−0.0006 (17)	0.0009 (17)	−0.019 (2)
C4	0.042 (2)	0.084 (3)	0.038 (2)	0.007 (2)	−0.0024 (17)	−0.0061 (19)
C5	0.040 (2)	0.072 (2)	0.0331 (18)	0.0042 (18)	0.0068 (15)	0.0042 (17)
C6	0.0321 (17)	0.0477 (17)	0.0295 (16)	0.0008 (14)	0.0061 (13)	−0.0046 (14)
C7	0.0331 (17)	0.0390 (15)	0.0311 (16)	0.0017 (13)	0.0059 (13)	0.0005 (12)
C8	0.0419 (19)	0.0414 (16)	0.0268 (15)	−0.0006 (14)	0.0095 (13)	−0.0013 (13)
C9	0.0356 (17)	0.0369 (15)	0.0333 (16)	0.0004 (13)	0.0124 (13)	0.0005 (12)
C10	0.0341 (17)	0.0321 (14)	0.0296 (15)	0.0013 (12)	0.0055 (13)	0.0027 (12)
C11	0.0377 (18)	0.0340 (14)	0.0264 (15)	0.0012 (13)	0.0056 (13)	0.0014 (12)
C12	0.0354 (17)	0.0430 (16)	0.0308 (16)	0.0004 (13)	0.0094 (13)	0.0000 (13)
C13	0.0360 (17)	0.0425 (16)	0.0256 (15)	−0.0026 (13)	0.0068 (13)	0.0007 (12)
C14	0.040 (2)	0.069 (2)	0.0356 (18)	0.0120 (18)	0.0085 (15)	0.0064 (17)
C15	0.048 (2)	0.107 (4)	0.031 (2)	0.016 (2)	0.0018 (17)	0.011 (2)
C16	0.056 (2)	0.095 (3)	0.0243 (17)	−0.003 (2)	0.0115 (16)	−0.0065 (19)
C17	0.057 (3)	0.081 (3)	0.040 (2)	0.014 (2)	0.0156 (18)	−0.0076 (19)
C18	0.050 (2)	0.061 (2)	0.0320 (18)	0.0145 (18)	0.0047 (15)	−0.0016 (16)
C19	0.0379 (18)	0.0423 (17)	0.0318 (16)	−0.0030 (14)	0.0070 (13)	0.0006 (13)
C20	0.0299 (17)	0.055 (2)	0.044 (2)	0.0015 (15)	0.0064 (15)	0.0005 (16)
C21	0.0341 (18)	0.0487 (18)	0.0388 (18)	0.0025 (14)	0.0085 (14)	0.0002 (15)
C22	0.0376 (19)	0.0495 (18)	0.0340 (17)	0.0020 (15)	0.0103 (14)	0.0016 (14)
C23	0.041 (2)	0.0477 (18)	0.048 (2)	0.0005 (16)	0.0061 (16)	−0.0040 (16)
C24	0.048 (2)	0.0403 (17)	0.051 (2)	−0.0066 (16)	0.0073 (17)	−0.0069 (16)
C25	0.045 (2)	0.0394 (17)	0.0386 (18)	0.0044 (14)	0.0112 (15)	−0.0018 (14)
C26	0.0398 (18)	0.0420 (16)	0.0336 (17)	−0.0008 (14)	0.0137 (14)	0.0026 (13)
C27	0.046 (2)	0.0360 (15)	0.0387 (18)	0.0007 (14)	0.0164 (15)	0.0025 (13)
C28	0.054 (2)	0.089 (3)	0.038 (2)	−0.007 (2)	0.0220 (18)	−0.008 (2)
C29	0.070 (3)	0.039 (2)	0.089 (4)	0.002 (2)	−0.011 (3)	0.001 (2)

### Geometric parameters (Å, °)

**Table:**

Br1—C26	1.882 (4)	C13—C14	1.384 (5)
F1—C3	1.370 (5)	C14—C15	1.390 (6)
F2—C16	1.370 (4)	C14—H14A	0.9300
O1—C9	1.378 (4)	C15—C16	1.344 (6)
O1—C28	1.427 (5)	C15—H15A	0.9300
O2—C19	1.218 (4)	C16—C17	1.351 (6)
O3—C25	1.362 (4)	C17—C18	1.392 (5)
O3—C29	1.422 (5)	C17—H17A	0.9300
C1—C2	1.379 (6)	C18—H18A	0.9300
C1—C6	1.390 (5)	C19—C20	1.468 (5)
C1—H1A	0.9300	C20—C21	1.327 (5)
C2—C3	1.360 (6)	C20—H20A	0.9300
C2—H2A	0.9300	C21—C22	1.479 (5)
C3—C4	1.369 (7)	C21—H21A	0.9300
C4—C5	1.383 (6)	C22—C23	1.380 (5)
C4—H4A	0.9300	C22—C27	1.394 (5)
C5—C6	1.392 (5)	C23—C24	1.398 (5)
C5—H5A	0.9300	C23—H23A	0.9300
C6—C7	1.485 (5)	C24—C25	1.366 (5)
C7—C12	1.386 (5)	C24—H24A	0.9300
C7—C8	1.398 (5)	C25—C26	1.398 (5)
C8—C9	1.379 (5)	C26—C27	1.386 (5)
C8—H8A	0.9300	C27—H27A	0.9300
C9—C10	1.391 (4)	C28—H28A	0.9600
C10—C11	1.394 (5)	C28—H28B	0.9600
C10—C19	1.522 (5)	C28—H28C	0.9600
C11—C12	1.394 (5)	C29—H29A	0.9600
C11—C13	1.498 (4)	C29—H29B	0.9600
C12—H12A	0.9300	C29—H29D	0.9600
C13—C18	1.380 (5)		
			
C9—O1—C28	117.3 (3)	C15—C16—F2	118.5 (4)
C25—O3—C29	117.3 (3)	C17—C16—F2	118.5 (4)
C2—C1—C6	122.0 (4)	C16—C17—C18	118.0 (4)
C2—C1—H1A	119.0	C16—C17—H17A	121.0
C6—C1—H1A	119.0	C18—C17—H17A	121.0
C3—C2—C1	118.1 (4)	C13—C18—C17	121.4 (4)
C3—C2—H2A	120.9	C13—C18—H18A	119.3
C1—C2—H2A	120.9	C17—C18—H18A	119.3
C2—C3—C4	122.5 (4)	O2—C19—C20	120.3 (3)
C2—C3—F1	118.5 (4)	O2—C19—C10	120.5 (3)
C4—C3—F1	119.0 (4)	C20—C19—C10	119.3 (3)
C3—C4—C5	119.0 (4)	C21—C20—C19	124.3 (3)
C3—C4—H4A	120.5	C21—C20—H20A	117.8
C5—C4—H4A	120.5	C19—C20—H20A	117.8
C4—C5—C6	120.7 (4)	C20—C21—C22	128.0 (4)
C4—C5—H5A	119.7	C20—C21—H21A	116.0
C6—C5—H5A	119.7	C22—C21—H21A	116.0
C1—C6—C5	117.8 (3)	C23—C22—C27	118.2 (3)
C1—C6—C7	120.8 (3)	C23—C22—C21	119.9 (3)
C5—C6—C7	121.3 (3)	C27—C22—C21	121.9 (3)
C12—C7—C8	118.4 (3)	C22—C23—C24	121.6 (4)
C12—C7—C6	122.0 (3)	C22—C23—H23A	119.2
C8—C7—C6	119.6 (3)	C24—C23—H23A	119.2
C9—C8—C7	119.9 (3)	C25—C24—C23	119.9 (3)
C9—C8—H8A	120.1	C25—C24—H24A	120.0
C7—C8—H8A	120.1	C23—C24—H24A	120.0
O1—C9—C8	122.6 (3)	O3—C25—C24	125.4 (3)
O1—C9—C10	115.6 (3)	O3—C25—C26	115.3 (3)
C8—C9—C10	121.7 (3)	C24—C25—C26	119.3 (3)
C9—C10—C11	118.9 (3)	C27—C26—C25	120.5 (3)
C9—C10—C19	118.2 (3)	C27—C26—Br1	119.5 (3)
C11—C10—C19	122.7 (3)	C25—C26—Br1	120.0 (3)
C10—C11—C12	119.1 (3)	C26—C27—C22	120.4 (3)
C10—C11—C13	122.9 (3)	C26—C27—H27A	119.8
C12—C11—C13	118.0 (3)	C22—C27—H27A	119.8
C7—C12—C11	122.0 (3)	O1—C28—H28A	109.5
C7—C12—H12A	119.0	O1—C28—H28B	109.5
C11—C12—H12A	119.0	H28A—C28—H28B	109.5
C18—C13—C14	118.0 (3)	O1—C28—H28C	109.5
C18—C13—C11	120.1 (3)	H28A—C28—H28C	109.5
C14—C13—C11	121.8 (3)	H28B—C28—H28C	109.5
C13—C14—C15	120.5 (4)	O3—C29—H29A	109.5
C13—C14—H14A	119.8	O3—C29—H29B	109.5
C15—C14—H14A	119.8	H29A—C29—H29B	109.5
C16—C15—C14	119.1 (4)	O3—C29—H29D	109.5
C16—C15—H15A	120.5	H29A—C29—H29D	109.5
C14—C15—H15A	120.5	H29B—C29—H29D	109.5
C15—C16—C17	123.0 (4)		
			
C6—C1—C2—C3	1.4 (7)	C12—C11—C13—C14	129.0 (4)
C1—C2—C3—C4	−1.4 (7)	C18—C13—C14—C15	0.2 (6)
C1—C2—C3—F1	178.8 (4)	C11—C13—C14—C15	−175.6 (4)
C2—C3—C4—C5	−0.1 (7)	C13—C14—C15—C16	−0.3 (7)
F1—C3—C4—C5	179.7 (4)	C14—C15—C16—C17	0.1 (8)
C3—C4—C5—C6	1.5 (6)	C14—C15—C16—F2	−179.9 (5)
C2—C1—C6—C5	−0.1 (6)	C15—C16—C17—C18	0.1 (8)
C2—C1—C6—C7	−176.8 (4)	F2—C16—C17—C18	−179.8 (5)
C4—C5—C6—C1	−1.4 (6)	C14—C13—C18—C17	0.0 (6)
C4—C5—C6—C7	175.3 (4)	C11—C13—C18—C17	175.9 (4)
C1—C6—C7—C12	−32.0 (5)	C16—C17—C18—C13	−0.2 (7)
C5—C6—C7—C12	151.4 (4)	C9—C10—C19—O2	108.8 (4)
C1—C6—C7—C8	146.0 (4)	C11—C10—C19—O2	−66.6 (4)
C5—C6—C7—C8	−30.6 (5)	C9—C10—C19—C20	−71.9 (4)
C12—C7—C8—C9	2.5 (5)	C11—C10—C19—C20	112.7 (4)
C6—C7—C8—C9	−175.6 (3)	O2—C19—C20—C21	165.2 (4)
C28—O1—C9—C8	1.9 (5)	C10—C19—C20—C21	−14.2 (5)
C28—O1—C9—C10	−176.1 (3)	C19—C20—C21—C22	−173.6 (3)
C7—C8—C9—O1	−177.7 (3)	C20—C21—C22—C23	176.7 (4)
C7—C8—C9—C10	0.2 (5)	C20—C21—C22—C27	−1.7 (6)
O1—C9—C10—C11	175.0 (3)	C27—C22—C23—C24	0.7 (6)
C8—C9—C10—C11	−3.0 (5)	C21—C22—C23—C24	−177.8 (4)
O1—C9—C10—C19	−0.5 (4)	C22—C23—C24—C25	0.9 (6)
C8—C9—C10—C19	−178.5 (3)	C29—O3—C25—C24	−5.3 (6)
C9—C10—C11—C12	3.0 (4)	C29—O3—C25—C26	174.8 (4)
C19—C10—C11—C12	178.4 (3)	C23—C24—C25—O3	177.1 (4)
C9—C10—C11—C13	−178.6 (3)	C23—C24—C25—C26	−3.0 (6)
C19—C10—C11—C13	−3.2 (5)	O3—C25—C26—C27	−176.6 (3)
C8—C7—C12—C11	−2.4 (5)	C24—C25—C26—C27	3.5 (5)
C6—C7—C12—C11	175.6 (3)	O3—C25—C26—Br1	3.6 (4)
C10—C11—C12—C7	−0.4 (5)	C24—C25—C26—Br1	−176.3 (3)
C13—C11—C12—C7	−178.9 (3)	C25—C26—C27—C22	−1.9 (5)
C10—C11—C13—C18	134.8 (4)	Br1—C26—C27—C22	177.9 (3)
C12—C11—C13—C18	−46.8 (5)	C23—C22—C27—C26	−0.2 (5)
C10—C11—C13—C14	−49.4 (5)	C21—C22—C27—C26	178.3 (3)

### Hydrogen-bond geometry (Å, °)

**Table:**

*D*—H···*A*	*D*—H	H···*A*	*D*···*A*	*D*—H···*A*
C29—H29B···O2^i^	0.96	2.41	3.303 (6)	155

Symmetry codes: (i) −*x*+1/2, *y*−1/2, −*z*+3/2.

## References

[bb1] Bruker (2009). *APEX2*, *SAINT* and *SADABS.* Bruker AXS Inc., Madison, Wiscosin, USA.

[bb2] Fun, H.-K., Hemamalini, M., Samshuddin, S., Narayana, B. & Yathirajan, H. S. (2010*a*). *Acta Cryst.* E**66**, o582–o583. 10.1107/S1600536810004435 PMC2983722 21580348

[bb3] Fun, H.-K., Hemamalini, M., Samshuddin, S., Narayana, B. & Yathirajan, H. S. (2010*b*). *Acta Cryst.* E**66**, o864–o865. 10.1107/S1600536810009414 PMC2983895 21580687

[bb4] Sheldrick, G. M. (2008). *Acta Cryst.* A**64**, 112–122. 10.1107/S0108767307043930 18156677

[bb5] Spek, A. L. (2009). *Acta Cryst.* D**65**, 148–155. 10.1107/S090744490804362X PMC2631630 19171970

